# A comparison of four different imaging modalities – Conventional, cross polarized, infra-red and ultra-violet in the assessment of childhood bruising

**DOI:** 10.1016/j.jflm.2018.07.015

**Published:** 2018-10

**Authors:** L. Trefan, C. Harris, S. Evans, D. Nuttall, S. Maguire, A.M. Kemp

**Affiliations:** a Division of Population Medicine, School of Medicine, Cardiff University, Heath Park, Cardiff, CF14 4YS, UK; b Chief Clinical Photographer, Dental Photography, School of Dentistry, College of Biomedical and Life Sciences, Cardiff University, Heath Park, Cardiff, CF14 4XY, UK

**Keywords:** Bruise descriptors, Conventional imaging, Cross polarized imaging, Infrared imaging, Ultra-violet imaging, Image J

## Abstract

**Background:**

It is standard practice to image concerning bruises in children. We aim to compare the clarity and measurements of bruises using cross polarized, infra-red (IR) and ultra-violet (UV) images to conventional images.

**Methods:**

Children aged <11 years with incidental bruising were recruited. Demographics, skin and bruise details were recorded. Bruises were imaged by standard protocols in conventional, cross-polarized, IR and UV lights. Bruises were assessed in vivo for contrast, uniformity and diffuseness, and these characteristics were then compared across image modalities. Color images (conventional, cross polarized) were segmented and measured by ImageJ. Bruises of grey scale images (IR, UV) were measured by a ‘plug in’ of ImageJ. The maximum and minimum Feret's diameter, area and aspect ratio, were determined. Comparison of measurements across imaging modalities was conducted using Wilcoxon rank sum tests and modified Bland-Altman graphs. Significance was set at p < 0.05.

**Results:**

Twenty five children had 39 bruises. Bruises that were of low contrast, i.e. difficult to distinguish from surrounding skin, were also more diffuse, and less uniformity in vivo. Low contrast bruises were best seen on conventional and cross-polarized images and less distinctive on IR and UV images. Of the 19 bruises visible in all modalities, the only significant difference was maximum and minimum Feret's diameters and area were smaller on IR compared to conventional images. Aspect ratios were not affected by the modality.

**Conclusions:**

Conventional and cross-polarized imaging provides the most consistent bruise measurement, particularly in bruises that are not easily distinguished from surrounding skin visually.

## Highlights

• Diffuse bruises may be measured on conventional & cross polarized imaging.
• Infrared or ultraviolet imaging may not show bruises which are difficult to see in vivo.
• Measurements of bruises using infrared imaging may be smaller than other modalities.

## 1 Introduction

Bruising is the most common manifestation of physical child abuse^1^ and may indicate significant underlying injuries. Certain patterns of bruising are more characteristic of abusive than unintentional injury^2^ Crucial to any assessment of bruising that may indicate child abuse is an accurate recording of the bruising found.

At present, clinical recording relies on digital imaging techniques.^3^ The appearance of a bruise varies according to the amount of hemoglobin visible in the indurated area followed by bio-degradational products resulting from the healing process. These changes may affect the absorbance and fluorescence properties of the skin.^4^ In order to visualize a bruise in the most effective way, different photographic imaging methods have been suggested, including cross polarized, infrared (IR) and ultraviolet (UV) imaging.^5^, ^6^, ^7^, ^8^

Conventional images are sometimes impaired by spurious light reflectance from the skin, caused by electronic flash. Cross polarized filters have been proposed to reduce this, by removing both the glare from reflected light in images, and the shine that sweat and oil in the skin produce.^5^ Cross polarized filters also enhance visual detail, and improve the definition of bruise margins, as the wavelength of reflected light is lengthened when it penetrates the skin's surface, heightening the bruise color and contrast.^9^ A previous study by Lawson et al. (2011),^10^ found that clinicians preferred cross polarized imaging when assessing images of bruises for boundary, color, size, shape and absence of light reflectance.

The longer wavelengths of infrared (IR) imaging may provide additional information about injuries below the surface of the skin, as they can penetrate relatively deeper in the skin, up to 3 mm,^11^ and have been demonstrated to be of benefit, in combination with color images, when analyzing traumatic injuries.^12^ This could be of particular value when imaging bruises on children with dark skin, as the level of penetration of IR waves, may cancel out the effect of the higher level of melanin in the epidermal layer of skin,^5^ thus making the bruises easier to see.

The shallow penetration of ultraviolet (UV) light into human skin results in less scatter of the reflected rays, and therefore in greater surface detail, and because of its shorter wavelengths, it offers greater resolution.^13^ It seems that when visual signs begin to disappear, UV images may be able to reveal old injuries, or at least support the identification of suspected injuries.^13^, ^14^, ^15^ Glauche et al. (2015)^16^ found that UV imaging is significantly better than physical examination in identifying hematomas when injuries are older than 1 week, and that it can be used to identify trauma up to 31 weeks after it has occurred. This is of particular importance in identifying bruises in cases of suspected physical abuse, as the clinical examination may occur some considerable period of time after the abuse has occurred.^14^^,^^17^

In addition to the *method of imaging*, the *measurement technique* used can influence the accuracy and reliability of bruise measurements. This has been investigated by Harris et al. (2017),^18^ who compared a range of suggested measurement techniques, including ImageJ segmentation, the circle technique (proposed by Bennett et al. (2013)^19^) and photoshop measurement, to the current standard practice, ie in vivo measurements, in both conventional and cross polarized images. The most consistent electronic measurements of children's bruises were ImageJ segmented conventional images. ImageJ has been widely used for almost 30 years^20^ to facilitate a more objective analysis^21^ of medical images of different skin problems.^22^

Comparison of bruise images using different modalities in live humans, is sparse. Baker et al. (2013)^5^^,^^17^ compared the general appearance of bruises on white (conventional), cross polarized, IR, and UV images. The greatest contrast was noted with conventional, and cross polarized light, and reduced performance on darker skin. Quantitative comparisons of maximum diameters of bruises on images taken in alternate light sources including conventional, IR and UV in a pigskin model^23^ and in non-embalmed and embalmed cadavers^24^ have been carried out lately using Fiji^25^ distribution of ImageJ software.

This study aims to compare an in-vivo assessment of bruises to the features seen using four different imaging modalities, namely conventional, cross polarized, infra-red and ultra-violet. In addition, we will compare measurements of the same bruises taken across each of these modalities.

## 2 Methods

### 2.1 Recruitment

Children aged less than 11 years were recruited from the emergency department and paediatric clinics, dental hospital clinic, and the regional haemophilia center (one child with haemophilia included) in Cardiff and Vale University Health Board. Recruitment took place between May 2009 and October 2011.

Parents were made aware of the study through information and flyers given to them by staff, and posters within the department. Staff contacted the research nurse if potential participants were interested in taking part in the study. Parents were provided with written information sheets prior to signed consent. Where appropriate, the child was offered age specific written information sheets and assent forms. Participants received £5.00 to cover travel expenses and could withdraw up to 24 h following consent.

Approval was given by the Research Ethics Committee on 07/05/2009, IRAS number: 09/H0504/53.

### 2.2 Data collection

The age of participants, the number, causes and location of the bruise(s) were documented. The imaging was undertaken in a quiet clinical space, with a parent present and a paediatric trained research nurse. The child's skin color was recorded using the Fitzpatrick Scale,^26^ the research nurse (DN) assessed the bruise and described it by 1–5 scale for uniformity (1 = single, solid consistent, 5 = speckled, patchy inconsistent), diffuseness (1 = strongly evident clear boundary, 5 = highly diffuse, faint, fuzzy edges), and contrast (1 = bruise stands out clearly from the skin, 5 = bruise very hard to detect). The measurement of the maximum diameter of the bruise (to the nearest millimeter mm) using a standard metric paper tape measure (in vivo *diameter*) was recorded. All information was anonymized with a unique identifier (ID) for each bruise, and entered into a spreadsheet (MS Excel).

### 2.3 Photographic techniques

All bruises underwent imaging utilizing the four different modalities. A standardized protocol was followed by trained Medical Photography technician's. The methods used are those previously described,^10^ whereby a Nikon D90 single-lens reflex camera was fitted with a Nikon 105-mm f/2.8 Micro-Nikkor lens, set at magnifications of 1:5 and 1:7. For IR and UV imaging, a Nikon D90 camera modified by Advanced Camera Services Ltd (ACS, Unit 10, Linmore Court, Threxton Road Industrial Estate, Watton, Norfolk IP25 6NG) solely for IR and UV imaging was employed.

All photographs were acquired by an experienced medical photographer using standard 105 Micro-Nikkor, this lens was used due to availability.

Photographic distortion was minimized by taking the image at a right angle (90°) to the surface. The cameras were set to Adobe RGB color space, and all images were recorded in RAW format. Each subject–image set included an image of a GretagMacbeth Mini Color-Checker as a check on color balance and/or exposure. An American Board Forensic Odontology (ABFO) No. 2^27^ scale was included in each bruise photograph. For specific differences in protocol for each imaging modality we refer to Table 2 of Lawson et al. (2011).^10^

### 2.4 Image processing

RAW format images were further processed by Photoshop software version CS4 for quality checking and converted in the Tag Image File Format (TIFF). All image quantification processes were performed using ImageJ software (1.45s)^28^ and using this Tiff image format.

On color images (*conventional, cross polarized*) a polygon was drawn around the bruise margin and a separate measurement of the surrounding skin, to differentiate the bruise from the surrounding skin (Appendix 1). These images were segmented by using SIOX (Simple Interactive Object Extraction) plugin of Image J. SIOX uses an algorithm which identifies the objects based on cascades of foreground and background colors. SIOX contains very effective noise filters and morphological operators, whereby a complex image segmentation process can be carried out, resulting in a black and white image with the bruise separated as an object.^29^

On grey-scale images (*IR and UV*) after drawing a polygon along the visible border of the bruise, Abdominal Adipose Tissue Assessment (AATAP) plugin of ImageJ^30^ was used. This utilizes an adaptive active contour algorithm (ABSnake),^31^ which creates an optimized border between the bruise and surrounding skin. Despite modifications including setting a lower gradient threshold of 5,^30^ and increasing the number of iterations to 50 of AATP plugin, segmentation was not possible. Thus, the optimized boundary for the bruise as described above was measured.

Ultimately, in both the color and greyscale images for each bruise, a region of interest (ROI) of ImageJ was defined, either along the segmentation border, or the optimized border. This constituted the baseline data for the quantification of the images.^32^

The herein described measuring techniques were carried out by an experienced operator and had been previously tested on dozens of (randomized) images per modalities.

The bruise measurements performed were: maximum Feret's diameter, minimum Feret's diameter, area,^33^^,^^34^ and aspect ratio.^32^ Maximum and minimum Feret's diameters measure the maximum and minimum dimensions of a bruise,^34^ the aspect ratio reflects the ratio between maximum and minimum dimensions and thus indicates shape.^35^ These commonly used measures in image processing^33^^,^^34^ were calculated by the software in units of pixels. In order to convert the Feret's diameters into mm, and the area to millimetres squared (mm^2^) for analysis purposes, the metric ruler scale of ABFO was used in each image and measured in pixels, with the resolution of each image established as mm/pixel.

### 2.5 Statistical analysis

All statistical analyses were conducted by R-software version 3.1.1 package and RStudio version 0.98.1062.^36^ Determination as to whether data were normally distributed was carried out by Shapiro-Wilk test and visually by Q-Q plot.^37^ The Wilcoxon rank sum test^38^ and the Fisher exact test^38^ were used to measure association. Significance was set at p < 0.05 for all tests. Agreements between the different modalities were assessed by modified Bland-Altman graphs^39^ where instead of using the difference of a measurement of a modality to the relevant conventional measurement as y axis, this difference as percentage of their means was used.^40^ For the Bland-Altman graphs precision terms were also calculated^39^^,^^40^

## 3 Results

**Fig. 1:**
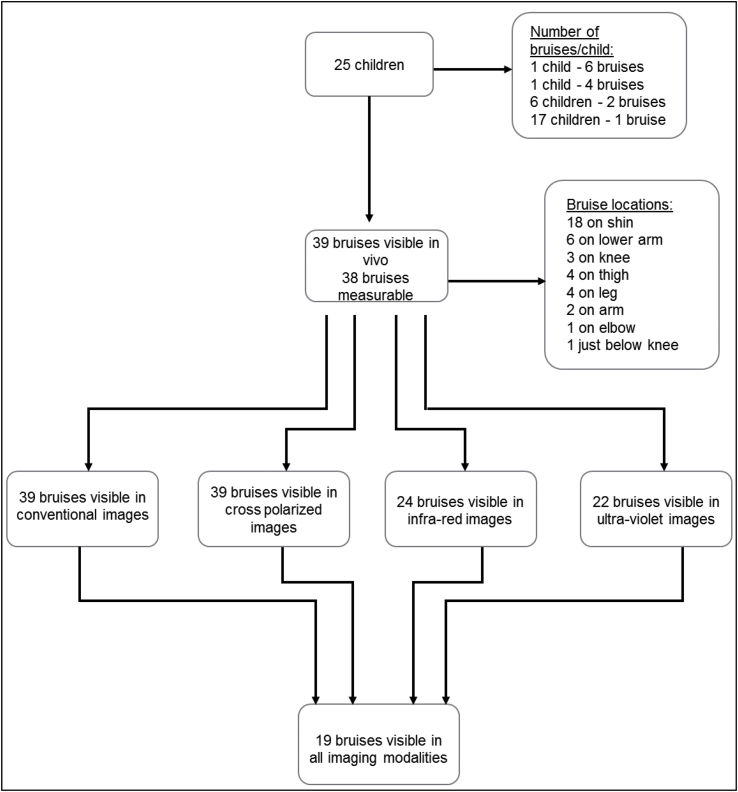
Details of 25 children recruited to the study, and the bruises visible on each modality.

The 39 bruises identified and imaged from the 25 children are detailed in Fig. 1. All children were Caucasian, 17 had type 2, and eight had type 3 skin on the Fitzpatrick Scale. All children were able to co-operate fully, and the time taken was approximately 5 min per bruise. One bruise could not be measured in vivo, although this bruise was detectable on both conventional and cross polarized images.

The in vivo assessments were combined to form two groups, comparing contrast to uniformity and diffuseness for those with a score of 1–3 to those with a score of 4–5 (Table 1). Bruises with lower contrast (not easily distinguished from surrounding skin) were also more diffuse, and significantly less uniform (p = 0.01).

**Table 1:** A comparison of the visible characteristics observed within 38 bruises on 25 children, according to their visible contrast, uniformity and diffuseness.

Contrast (score 1–5)	Uniformity		Diffuseness	
	1 to 3 (consistent)	4 to 5 (patchy,inconsistent)	1 to 3 (clear boundary)	4 to 5 (diffuse boundary)
**1 to 3** (clearly distinguishable)	7	5	6	6
**4 to 5** (difficult to detect)	6	21	3	24

The classification for each characteristic was defined as follows: **uniformity** (1 = single, solid consistent, 5 = speckled, patchy inconsistent), **diffuseness** (1 = strongly evident clear boundary, 5 = highly diffuse, faint, fuzzy edges), and **contrast** (1 = bruise stands out clearly from the skin, 5 = bruise very hard to detect). For analysis purposes, scores of 1–3 and 4–5 combined.

Fig. 2a, Fig. 2ba & b explore a comparison between the visible characteristics (contrast) and their clarity on each of the four imaging modalities. Among the 12 bruises which were easily distinguishable from surrounding skin in vivo, nine could be seen in all four modalities, while three were only visible on conventional and cross polarized images (Fig. 2a). The remaining 27 bruises which were less easily distinguishable from surrounding skin in vivo (contrast 4–5) showed a more varied picture, with only 10 seen in all four modalities and all 27 on conventional and cross polarized alone (Fig. 2b).

**Fig. 2a:**
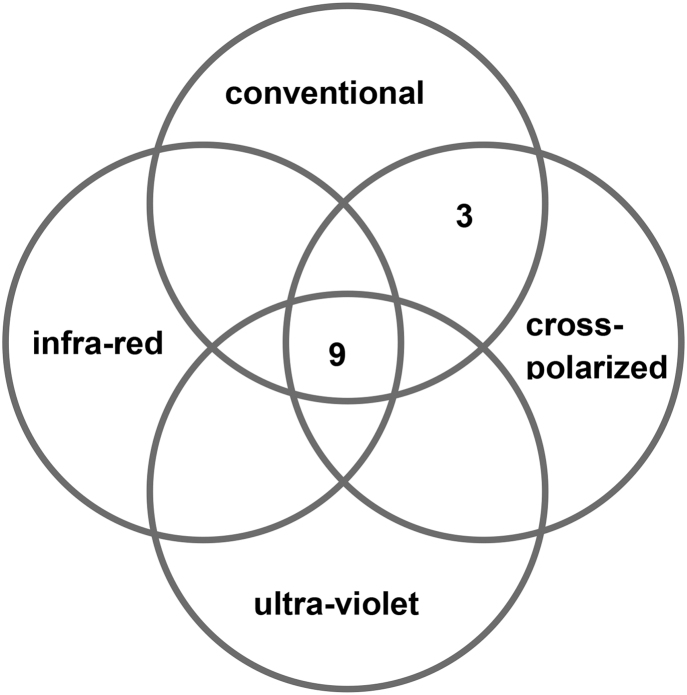
Bruises which are clearly visible (contrast 1–3), as seen on each of four imaging modalities: conventional, cross-polarized, infra-red and ultra-violet (n = 12).

**Fig. 2b:**
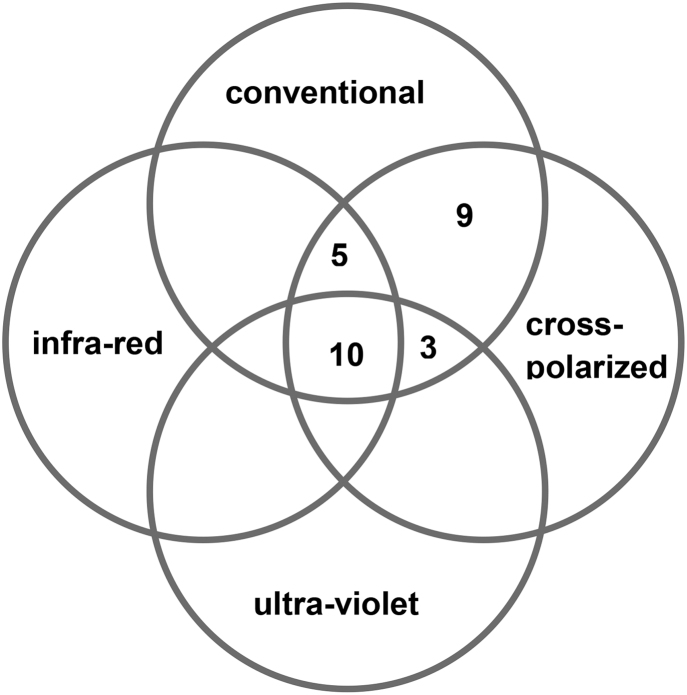
Bruises which are less clearly visible (contrast 4–5) as seen on each of four imaging modalities: conventional, cross polarized, infra-red and ultra-violet (n = 27).

Table 2 shows the descriptive statistics (mean, standard deviation) of the bruise images. The maximum and minimum Feret's diameters and area were significantly *smaller* on IR than on conventional images, with no significant differences between UV and cross polarized for the same comparisons. There were no significant differences between the *aspect ratios* when comparing cross polarized, IR or UV images to the conventional images (Table 2).

**Table 2:** The mean and standard deviation (SD) of bruise sizes, maximum and minimum Feret's diameter, area and aspect ratio of 19 bruises seen in all four imaging modalities: conventional, cross polarized, infra-red and ultra-violet.

Bruise descriptor	Conventional	Cross polarized	Infra-red	Ultra-violet
Maximum Feret's diameter (mm)	22.90 (12.49)	24.44, (13.61) (p = 0.66)	18.06*, (11.27) (p < 0.05)	21.95, (10.05) (p = 0.59)
Minimum Feret's diameter (mm)	14.41, (6.86)	15.21, (8.15) (p = 0.59)	11.67*, (7.86) (p = 0.03)	14.82, (7.64) (p = 0.61)
Area (mm^2^)	239.92, (232.18)	265.09, (257.73) (p = 0.63)	156.51*, (208.47) (p = 0.03)	233.57, (222.07) (p = 0.55)
Aspect ratio	1.68, (0.49)	1.73, (0.54) (p = 0.68)	1.75, (0.65) (p = 0.55)	1.63, (0.45) (p = 0.48)

* indicates a significant difference between the individual modality and conventional measurement by Wilcoxon rank sum test (p < 0.05). mm = millimeter. mm^2 =^millimeter.

In the more detailed analysis, the Bland-Altman graphs confirmed these findings, and showed that ultraviolet images had the widest range of diameters, and area, in comparison to conventional images (Appendix 2-5). The second widest range belonged to IR images considering minimum Feret's diameter and area for the same comparisons (Appendix 3 and Appendix 4). Range of aspect ratios had fallen by the same magnitude (Appendix 5). Two outliers can be seen on the Bland-Altman graphs (one on Appendix 2 A and one on Appendix 5 B). These outliers relate to unusually shaped bruises, which may have influenced their maximum diameter measurements. The variation between measurements showed random variability for all modalities.

These results indicate that cross polarized and UV images give a similar estimated size to that seen in a conventional image, but IR results in a smaller measurement. However, it appears that the overall ratios of the bruise dimensions on images remain unchanged in each of the four modalities, i.e. the shape is retained.

## 4 Discussion

With the increasing availability of different imaging modalities for use in forensic and clinical practice, it is important to determine their usefulness and limitations. Previous work has primarily used animals or cadavers, in contrast to live children as used in this study. We have shown that bruises which are less clearly seen in vivo are visualized on conventional and cross polarized imaging, but may not be visible on ultra-violet or infra-red imaging. In addition, standardized measurements of bruise size and shape using a computerized analysis of images, indicates that bruises recorded by IR may appear smaller than they are on conventional images, although they retain the same shape.

Utilizing conventional images as the standard imaging technique, it appears that cross-polarized and ultraviolet images give unbiased estimates of dimensions (maximum and minimum size, and area) of visible bruises. We chose to analyze four different descriptors in the computer aided bruise measurements, in comparison to previous studies that measured only the maximum dimensions of bruises in different image wavelengths. Olds et al. (2016)^23^ compared maximum Feret's diameter of bruises in a pigskin model on conventional, UV and IR images. Significant differences were found between the diameters on UV and conventional images; on IR images these Feret's diameters were not measurable. Olds et al. (2017)^24^ found significant differences of Feret's diameters measured on conventional and UV images, in contrast to our study. There may be a variety of reasons for this, including the photographic techniques or image measuring method applied, or the use of pigskin or cadaver's skin in contrast to bruises on living children. It is interesting to note that these previous studies alluded to “areas that were not histologically confirmed as bruises were also detected”. Recent work by Mimasaka et al.^17^ exploring the use of violet and UV light to visualize older bruises in live children observed that bruises were visible up to an average of 1.1 months using UV and 2.1 months using violet light. However, although measurements of bruises were given, no details were provided as to how these measurements were conducted, or any verification of their measurement techniques.

In assessing bruises in children with suspected abuse, it is important to have a reliable method of delineating and measuring the bruise, as this becomes a vital part of the forensic evaluation. If one is to determine the plausibility of the mechanism proposed, or match injuries found to a proposed mechanism, this can be more reliably achieved from a well delineated injury than one with a diffuse edge. It was apparent during this work that bruises with less easily defined margins could be more clearly measured using conventional and cross-polarized imaging. Related to these two modalities, the magnitude of delineation of computer aided techniques considering bruise maximum size measurements over in vivo observation, were discussed in the authors' previous work.^18^ Thus if one were to attempt to match a given patterned bruise to an implement such as a hairbrush, this may be more easily achieved using a combination of conventional and cross-polarized images, than by assessment with the naked eye.

From a technical viewpoint, we chose to use the SIOX plug-in to conduct segmentation of the color images, which has the potential advantage of integrating all of the necessary steps for full image processing. However, SIOX cannot segment any shape which has a ‘hole’ in it.^29^ This situation did not arise in the bruises studied here, thus we cannot comment on its usefulness in this situation. The difficulties encountered in attempting to segment the IR and UV images related to the inability of the system to clearly define a margin of the bruise. Baker et al. (2013)^5^ found significantly higher bruise contrast on conventional and cross polarized images then on IR or UV images on light toned skin, although there was no significant differences on darker skin tones. These findings may explain the difficulties we had segmenting IR and UV images. Therefore a boundary was established by completing an outline for the region of interest.

While other measurements can be chosen in Image J, such as perimeter, these were not used in this analysis. This was partly due to difficulties in segmentation that arose due to variability in the source data, due to artefacts in the structure of the segmented or established boundaries, rather than the structure of the actual bruise boundary. Additionally, perimeters which are calculated by different algorithms tend to show factorial dimensions which can make it difficult to establish a single number as a perimeter.^32^

As the data were not normally distributed, modified Bland-Altman graphs^40^ were created to highlight the differences between the individual modalities for each computer aided bruise descriptor. Due to the relatively small number of analyzed images, this method allowed for more detailed precision terms to be identified as well.^39^^,^^40^

Limitations to this study include the fact that all children were Caucasian, due to the low prevalence of ethnic minority children in our population. In addition, as only a single observer conducted the in vivo assessment, inter-class correlations could not be determined.^41^ Likewise, there were only 39 bruises available for analysis, and not all bruises were seen in each modality, however most studies in this field only relate to small study numbers^17^ which may reflect that challenges to recruitment and participation of young children and coordinating imaging of bruises that can resolve quickly to the naked eye over time. For forensic and clinical photographers employing the various modalities requires only a small amount of training. However, non-photographers may require more extensive training. All photographs were acquired using a previously described standard lens due to availability. The authors acknowledge that the quartz/fluorite lens may be a more appropriate lens when employing UV, as such, there was an inevitable reduction in quality in the UV images.

This study used images of same aged bruises in four modalities (conventional, cross polarized, IR, UV) and did not study how IR and UV techniques could extend visibility of bruises over time.

## 5 Conclusions

Given the potential forensic significance of bruises in children during child abuse evaluations, it is important to be aware that bruises of low contrast in vivo can be delineated, and reliably measured using conventional and cross polarized imaging. This study has demonstrated that it is possible to define the size of a bruise across conventional, cross polarized and UV imaging modalities using computer software. Accurate recording of bruises and precise measurements are relevant in conducting robust record keeping for any cases of suspected child abuse. Similarly, in any subsequent forensic analysis precise measurements are essential for proper matching techniques to potential implements used. As the techniques for identifying bruises of different sizes and ages are increasingly used in the assessment of possible child abuse, it is valuable to know the limitations of any measurements performed.
