# Supplementary material for: A comparison of four different imaging modalities – Conventional, cross polarized, infra-red and ultra-violet in the assessment of childhood bruising
Source: J Forensic Leg Med. 2018 Oct;59:30–5. doi: 10.1016/j.jflm.2018.07.015 (PMC6125673; doi:10.1016/j.jflm.2018.07.015)
Supplement: Appendix 1 [file mmc1.doc]

**Appendix 1**

**Detailed Methodology for segmentation and measurements of bruises on conventional and cross-Polarized images, using ImageJ software**

**Segmentation of conventional and cross-polarized images**

Segmentation of colour images can be done using the SIOX (Simple Interactive Object Extraction) plug-in of ImageJ. Eventually this plug-in produces a black and white image where the bruise is separated as an object.

SIOX using an algorithm which has a very effective noise filter and identifies the objects based on cascades of foreground and background colours. Therefore proper definition of foreground and background colours is crucial for the success of the segmentation.

**Step 1**: Start ImageJ. Open the image (Ctrl+O). Go to Plugins ⭢ SIOX Segmentation. The following will be displayed:


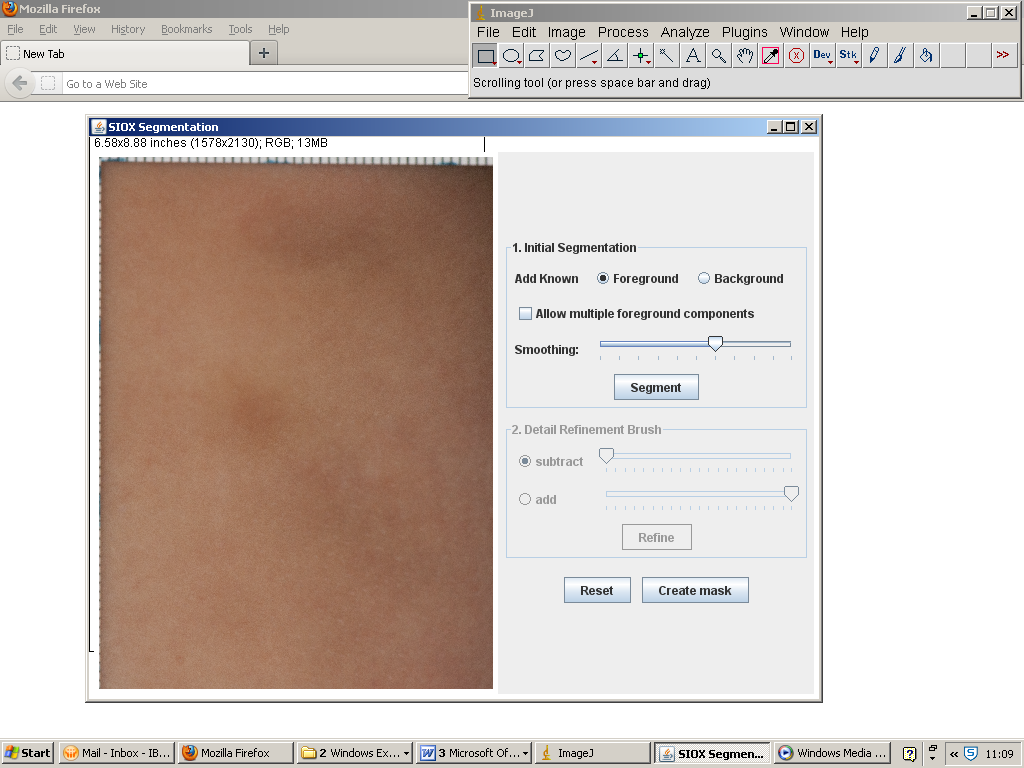


If more than one bruise was seen please click ‘Allow multiple foreground components’.

By pressing the ‘Shift’ button the additional bruise(s) can be defined as part of the ‘Foreground’. Please use ‘Freehand selections’ option when completing marking of different objects in all the following instructions.

**Step 2:** Mark ‘Foreground’, using the ‘Freehand selections’ option to cover the bruise to its borders.


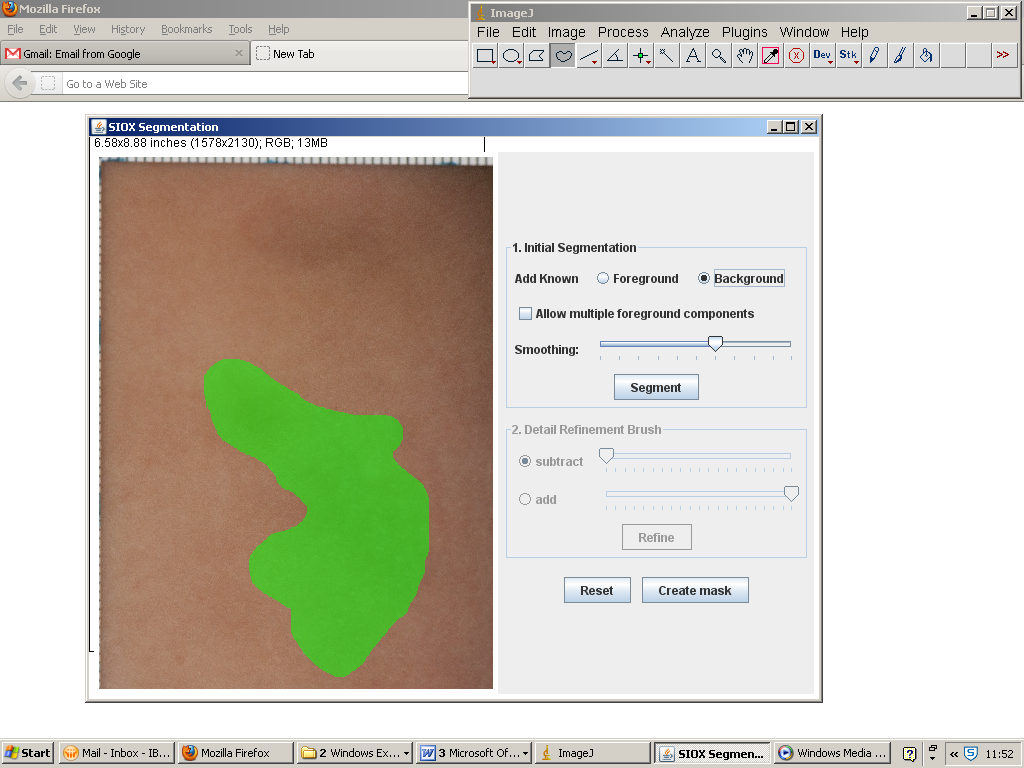


**Step 2a:** In the case of more than one bruise, mark all the separate objects as foregrounds:


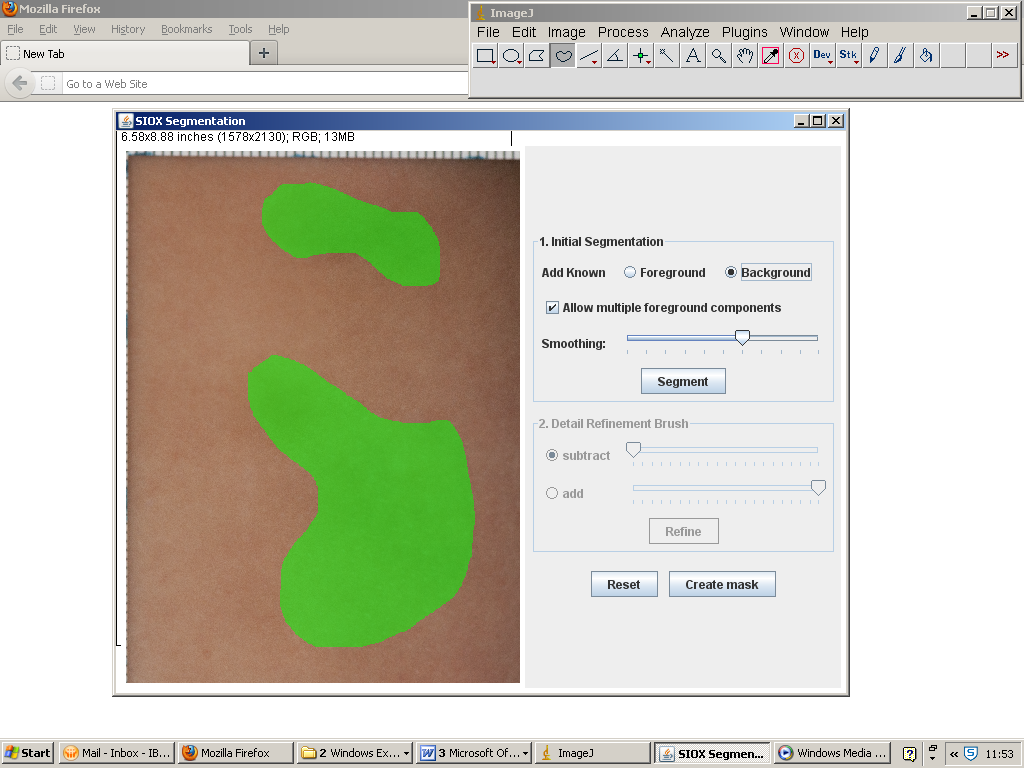


**Step 3:** Mark ‘Background’, by drawing as closely around the bruise(s) borders as possible, then drawing around the edge of the image, creating as large a connected area as possible (see thin green lines on image below).


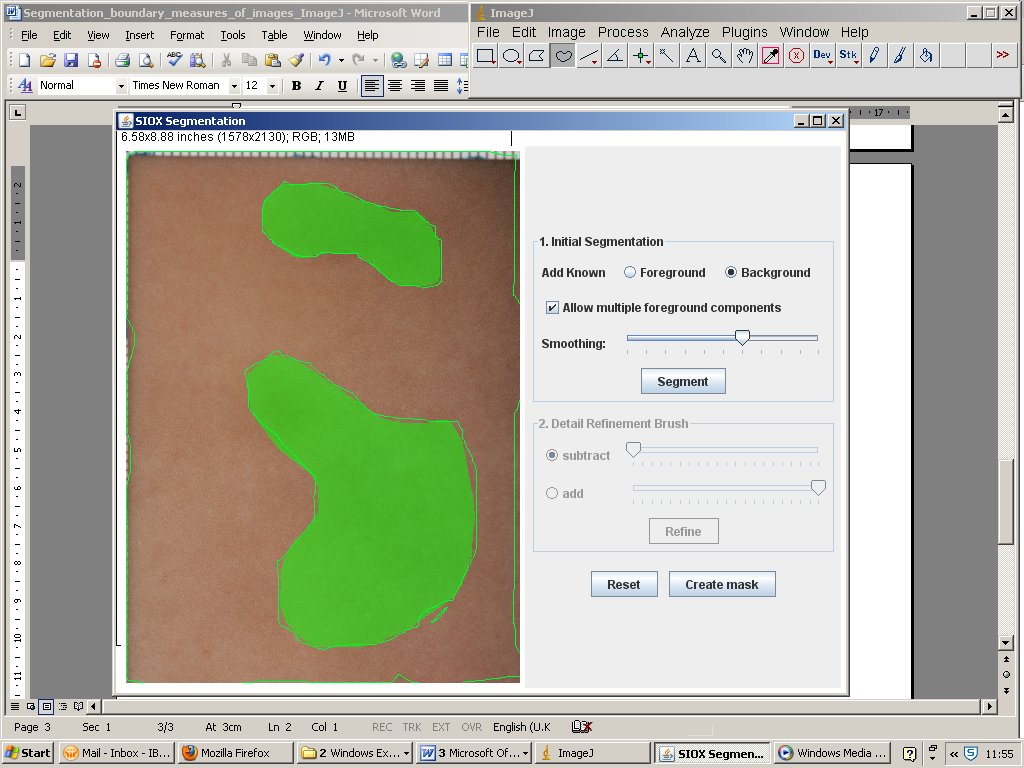


**Step 4:** Press ‘Segment’ and the result should look similar to the following picture:


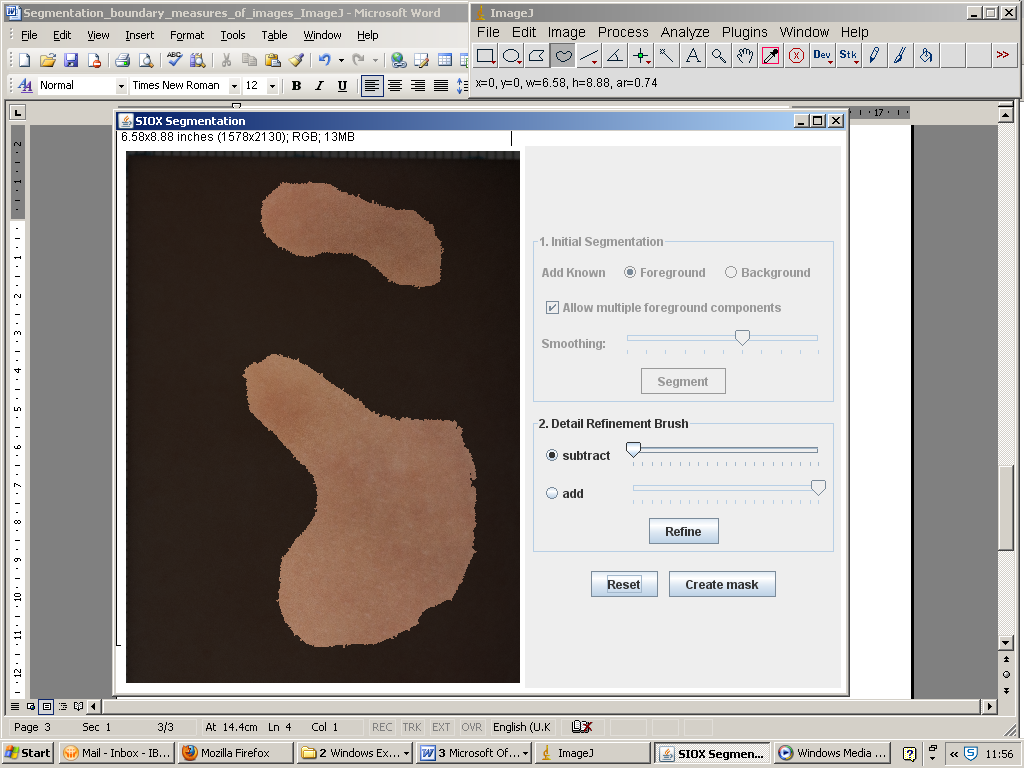


**Step 5:** If you are satisfied with the results (the entirety of the bruise(s) are shown and all background, non-bruised skin is not visible), press ‘Create mask’ and your segmented bruise(s) will be displayed as white objects on a black background.

Save the ‘Mask’ as a tiff file, by going to File ⭢ Save as ⭢ Image_name.**tiff**.


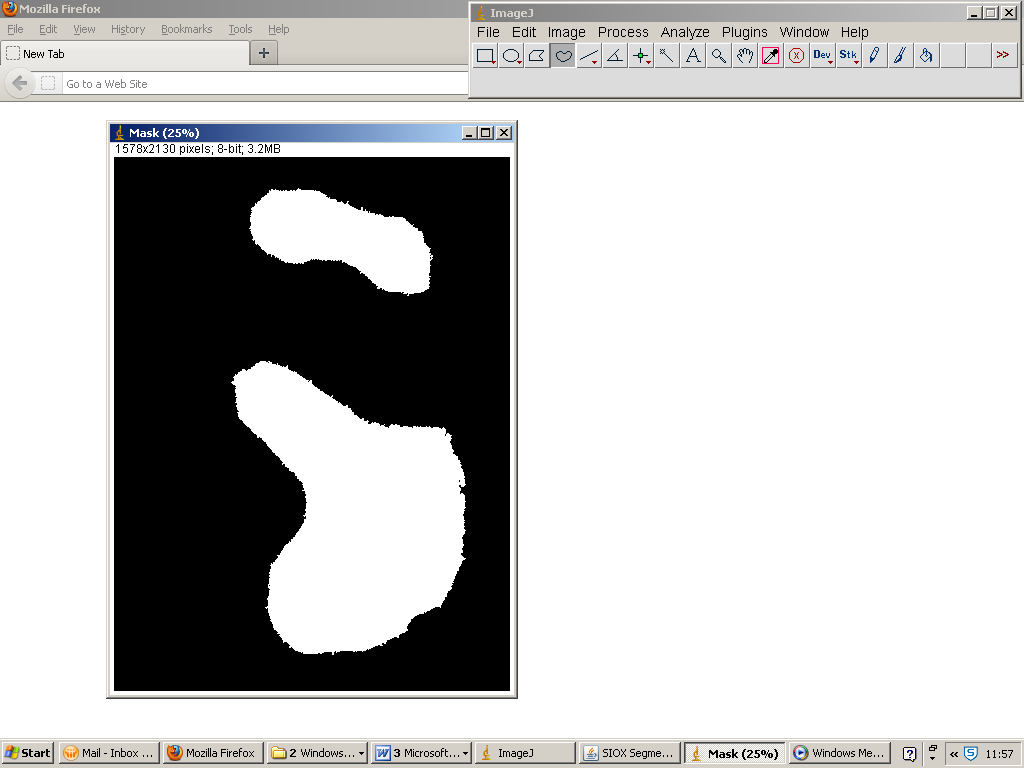


If you are **not satisfied** with the results, you can do the following:

On a **colour image**:

Press ‘Reset’ and, leaving the defined foreground and background, the colour rates can be changed to allow easier visualisation of the bruise, in order to confirm if you have segmented the correct areas, by tuning up/down on the ‘Smoothing’ button, if you are satisfied you can then go to Step 3.

If not, press ‘Reset’ and the process can be restarted, from Step 1 onwards, to allow new foreground and background boundaries to be defined.

On a **segmented, black & white image** (Step 3 & 4):

By clicking ‘Refine’, the foreground boundary can be ‘subtracted’, and background can be ‘added’, to allow more specific delineation of bruise borders.

**Further info on SIOX:**

More information, background and detailed references on SIOX can be found under the following link:

<http://fiji.sc/wiki/index.php/SIOX:_Simple_Interactive_Object_Extraction>

**Measuring the segmented images**

Before you start any measuring, you need to set which measurements of an object you want to determine.

**Setting the measurements of ImageJ:**

Please go to Analyze ⭢ Set Measurements and select which descriptors of an object you want to use as quantitative descriptors.


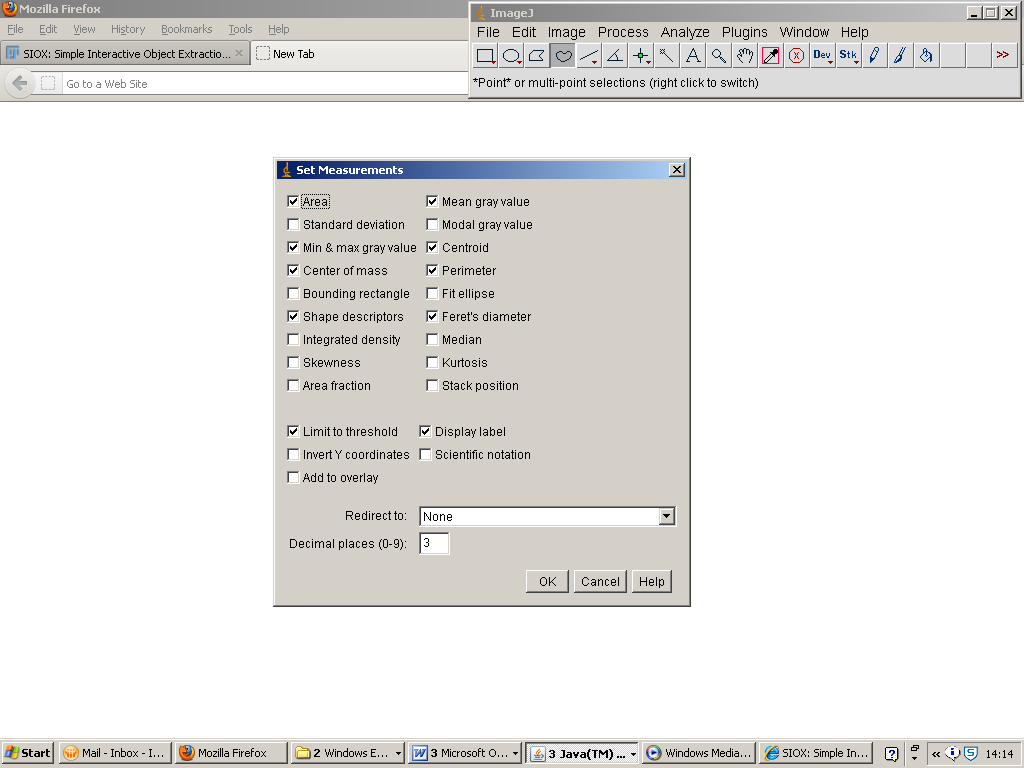


**Step 1**: Please open the image (Ctrl+O), and select the tiff file you wish to work with.


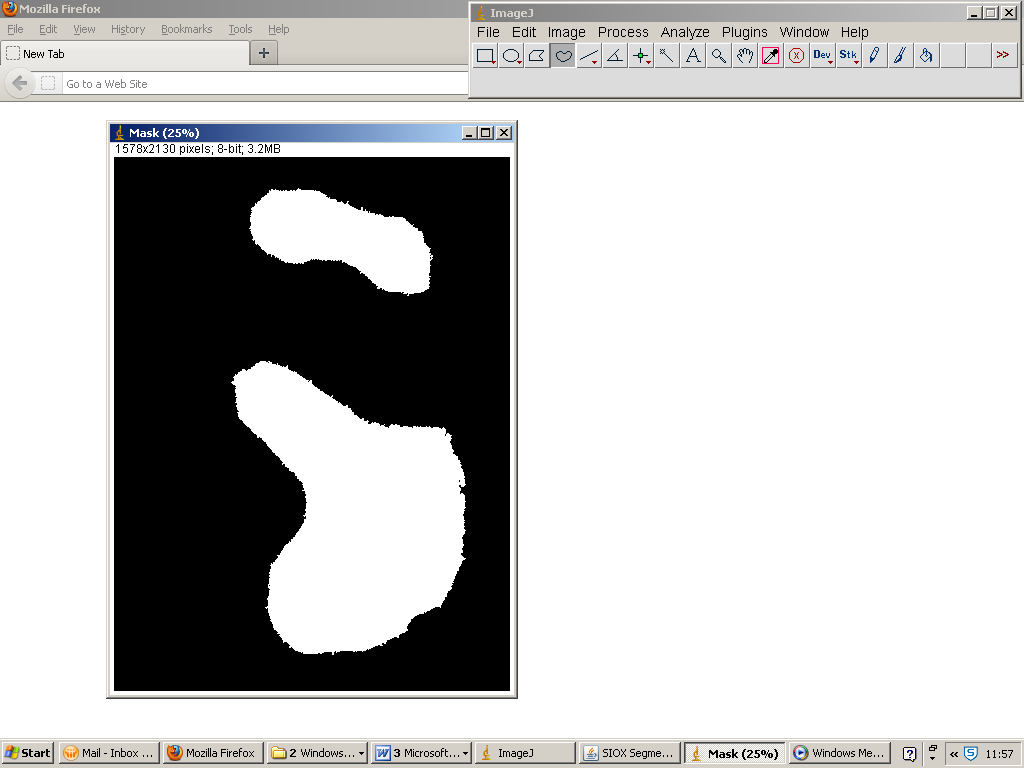


**Step 1:** If you had more than 1 object (bruise) please click ‘Flood Fill Tool’ on the ImageJ toolbar and fill in all objects except for one.


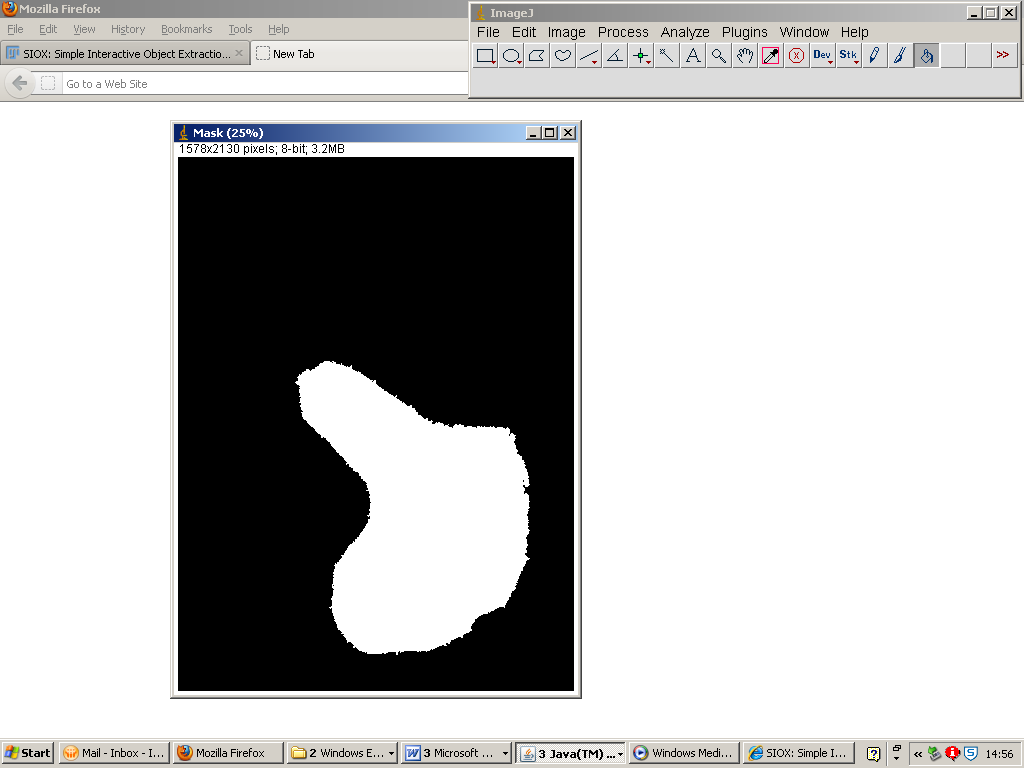


**Step 2:** Convert the image to get the object as black and the background as white, by pressing Ctrl+Shift+I, or clicking Edit ⭢ Invert.


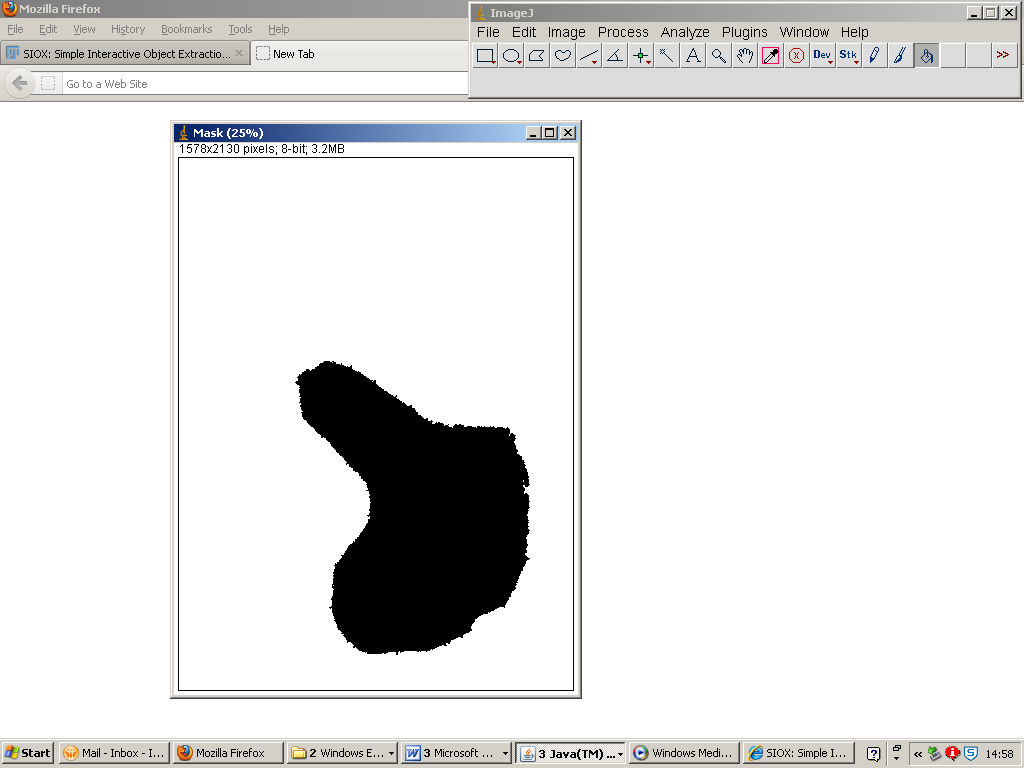


**Step 3:** Go to Edit ⭢ Selection ⭢ Create Selection. This selects the object to be measured and saved.
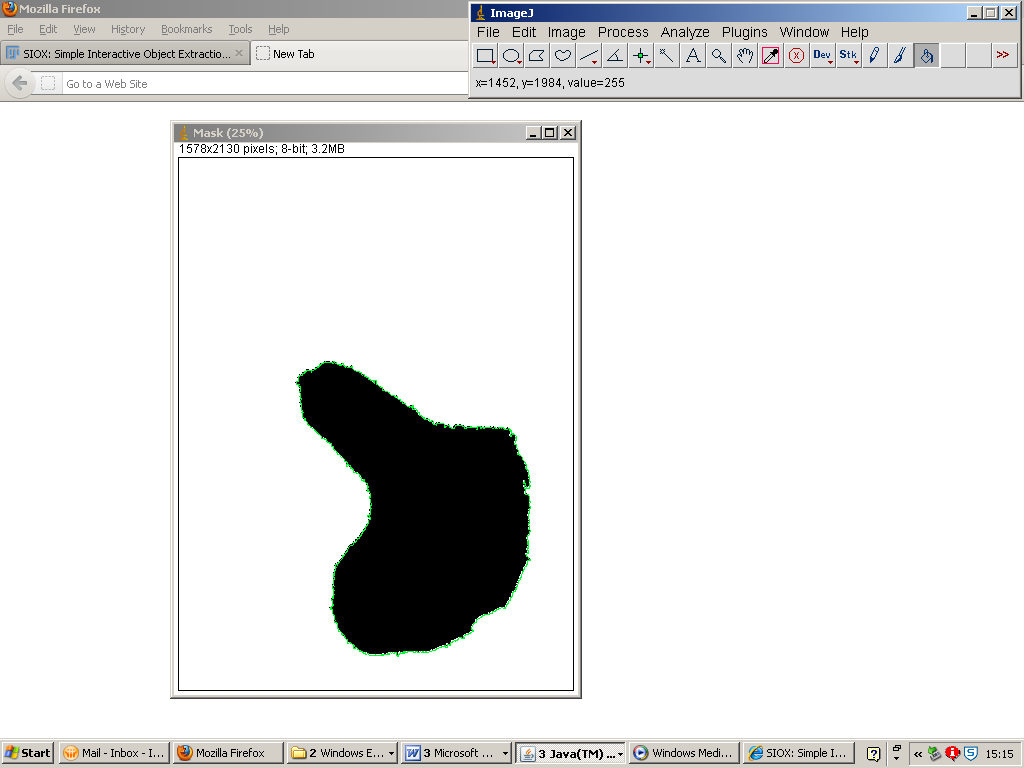


**To get measurement results:** Please press Ctrl+M, or go to Analyze ⭢ Measure, and you will get the results of measurement descriptors in pixel values (as selected prior to opening image). If this does not work correctly, you may need to go to Analyze ⭢ Ste Scale, and click ‘Click to Remove Scale’ on the pop-up window.

In the ‘Results’ pop-up window you can edit your results:

- Clear - clears all measurements
- Ctrl+X - cut only one row of results
- Ctrl+A - mark all results
- Ctrl+C - copy result(s).

After several measurements, the results can be saved by clicking File ⭢ Save As, the final file format is going to be tabulate delimited format, which can be directly handled by MS Excel.

**Saving the selection:** Please go Analyze ⭢ Tools ⭢ ROI Manager. In the pop-up window press ‘Add [t]’, a randomised number will appear. Click ‘Rename’ and in the pop-up window you can define a descriptive name for the selection. If you click ‘More >>’ in the ROI manager in the flashing window, then click ‘Save’ you can save your selection. It will have Selection_name.**roi** extension in your folder.

**Using a saved selection:** Go to Analyze ⭢ Tools ⭢ ROI Manager and click ‘More >>’ in the ROI manager in the flashing window, click ‘Open’ and your saved selection will be placed on your opened image. If you follow the previous ‘To get measurement results’ steps, as above, you will be able to measure your selected object on your image.

**Establishing spatial resolutions**

Knowing the spatial resolution of the each image is required to convert the above measurements from pixels to units of mm/cm or mm^2^/cm^2^.

Pixels alongside both legs of the ruler need to be measured because during the imaging process exact pixel vs. length sometimes cannot be established, and the imaged legs of the ruler may sometimes not be perpendicular to each other.

**Step 1:** Open the **original image** (Ctrl+O). Draw a horizontal line along the ruler to a pre-determined length in cm, as shown by the thin green line in the below image drawing to 3cm.


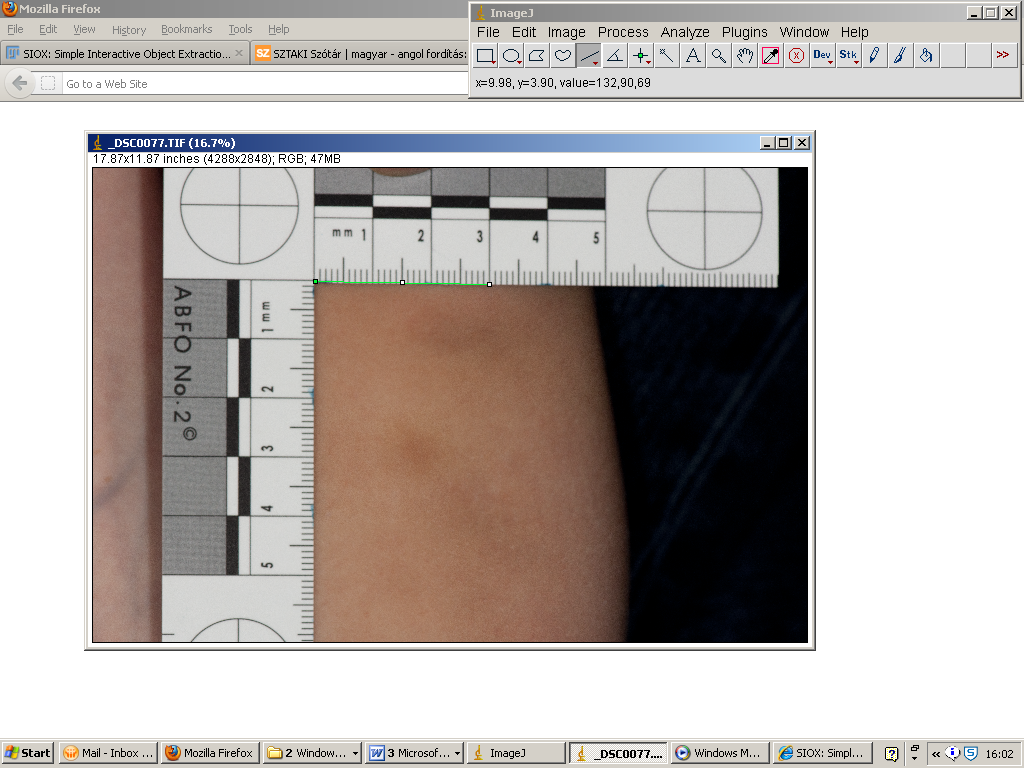


**Step 2:** Go to Analyze ⭢ Set Scale, in the pop-up window click ‘Click to Remove Scale’.

In this window you will have a number in the ‘Distance in pixels’ box, which is your drawn distance in pixels. Please record this value and the value on the ruler into a previously prepared sheet.

Repeat the above procedure on the vertical ruler and record these results into the sheet as well.

These combined pixel and cm results can then later be used to convert the measurements you have gained from the segmentation and measurement process, in pixels, into mm/cm or mm^2^/cm^2^.
