# Supplementary material for: A comparison of four different imaging modalities – Conventional, cross polarized, infra-red and ultra-violet in the assessment of childhood bruising
Source: J Forensic Leg Med. 2018 Oct;59:30–5. doi: 10.1016/j.jflm.2018.07.015 (PMC6125673; doi:10.1016/j.jflm.2018.07.015)

**Appendix 5**

Bland-Altman graphs of comparing aspect ratio (AR) (in percentage of means) of 19 bruises on cross polarized-, infrared (IR)- and ultraviolet (UV)- to conventional images. Middle (bold) line shows mean of the difference in percentage of means, with 95% confidence intervals (upper-, lower bold lines). Grey lines show their relevant precision terms.


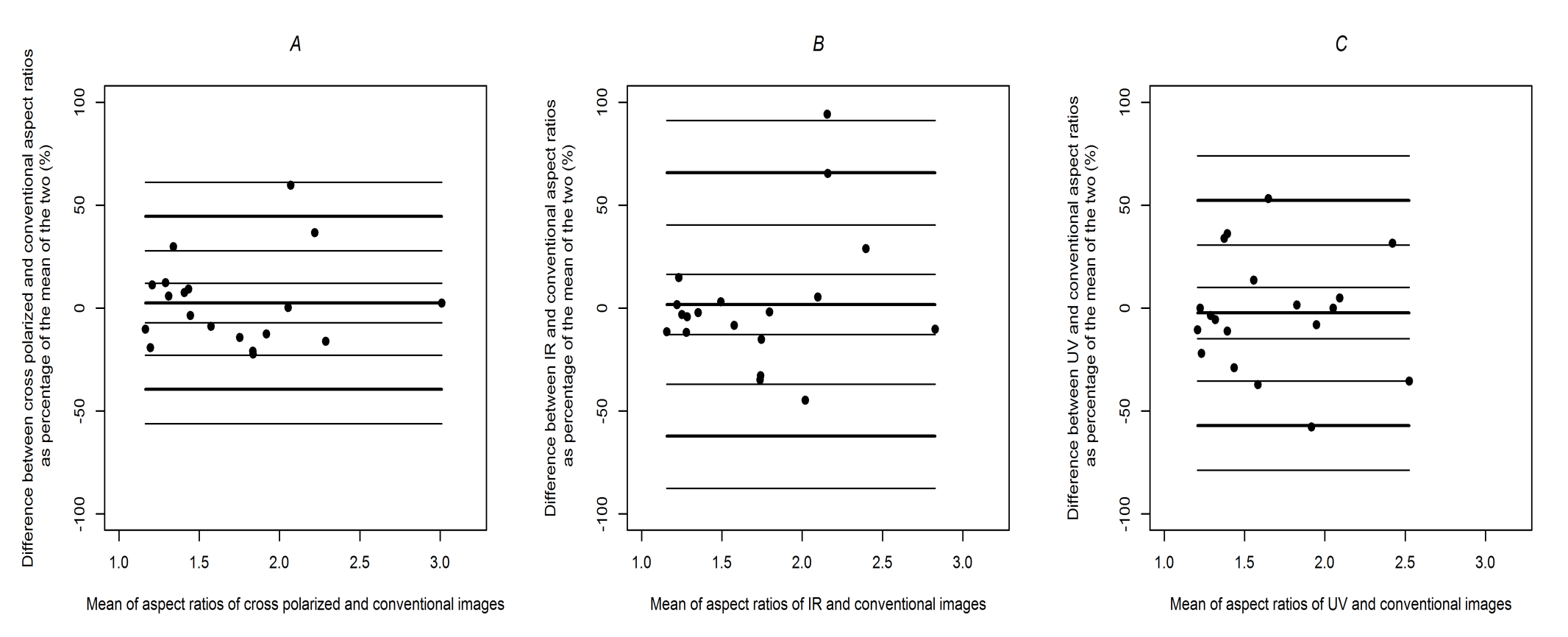

Supplement: Appendix 5 [file mmc5.docx]
